# Diazepam Loaded Solid Lipid Nanoparticles: *In Vitro* and *In Vivo* Evaluations

**DOI:** 10.34172/apb.2022.008

**Published:** 2020-09-08

**Authors:** Sara Faghihi, Mohammad Reza Awadi, Seyyedeh Elaheh Mousavi, Seyyed Mahdi Rezayat Sorkhabadi, Mandana Karboni, Shirzad Azarmi, Solmaz Ghaffari

**Affiliations:** ^1^Department of Pharmaceutics, Faculty of Pharmacy, Tehran Medical Sciences, Islamic Azad University, Tehran, Iran.; ^2^Department of Research and Development, Hakim Pharmaceutical Co, Tehran, Iran.; ^3^Department of Pharmacology, School of Medicine, Tehran University of Medical Sciences (TUMS), Tehran, Iran.; ^4^Department of Medical Nanotechnology, School of Advanced Sciences and Technology in Medicine, Tehran University of Medical Sciences (TUMS), Tehran, Iran.; ^5^Faculty of Pharmacy and Pharmaceutical Sciences, University of Alberta, Edmonton, Alberta, Canada.

**Keywords:** Blood distribution, Diazepam (DZP), Drug delivery, Solid Lipid Nanoparticles (SLN), Sustained release

## Abstract

***Purpose:*** To overcome the side effects of repetitive administration of diazepam (Dzp) besidesgaining benefits from sustaining release of the drug, which contributes to patient compliance,we concentrated on designing and preparing Dzp solid lipid nanoparticles (SLNs).

***Methods:*** Using cholesterol (CHOL), stearic acid (SA), and glycerol monostearate (GMS), SLNswere prepared by high shear homogenization technique coupled with sonication. Polysorbate80 (Tween 80) was used as a nonionic surfactant. After modification of prepared SLNs, particlesize, zeta potential, drug-loading efficiency, morphology, and scanning calorimetry, as well asrelease studies were conducted. To increase the stability of desired particles, freeze-drying bycryoprotectant was carried out. In the final stage, *In vivo* studies were performed by oral (PO)and intraperitoneal (IP) administrations to Wistar male rats.

***Results:*** Results indicated that optimized prepared particles were on average 150 nm diameterin spherical shape with 79.06 % loading efficiency and release of more than 85% of the loadeddrug in 24 hours. *In vivo* investigations also illustrated differences in blood distribution of Dzpafter loading this drug into SLNs.

***Conclusion:*** Based on the findings, it seems that drug delivery using SLNs could be anopportunity for solving complications of Dzp therapy in the future.

## Introduction

Diazepam (Dzp) belongs to a class of drugs called benzodiazepines, which enhances the effect of gamma-aminobutyric acid, a neurotransmitter that moderates the activity of nerve signals in the brain. Dzp has some side effects including drowsiness, fatigue, muscle weakness, and clumsiness.^1^ This may lead to reducing patient compliance when repetitive administration is needed. To overcome this problem, numerous studies have been carried out for sustaining the release of Dzp. Sharma et al^2^ tried to optimize Dzp-loaded poly (lactic-co-glycolic acid) nanoparticles to achieve delivery to the brain through intranasal administration. In their study, Dzp nanoparticles (DNP) were formulated by nano-precipitation technique and drug release was reported about 61% in 24 hours. Some researchers have also addressed a problem regarding the poor solubility of Dzp.^3^ Despite being lipophilic, Dzp is rapidly redistributed out of the brain.^2,4^ Dzp is poorly soluble in water, and its intravenous formulation has to be prepared using co-solvents such as propylene glycol (40%) and ethanol (10%) which, in turn, may result in adverse effects. Considering this issue, lipid nanoparticles could be applied as an interesting solution;^2,5^ as Đorđević et al^6^ formulated Dzp nanoemulsion with the aim of tackling the mentioned side effects. Bohrey et al^7^ sustained the release of Dzp for 9 hours by formulating polymer nanoparticles.

Cyclodextrin conjugated magnetic nanoparticles were also fabricated and designed by Cai et al,^8^ to prevent side effects of Dzp remaining in the body after multiday of usage. Some studies were reported on designing Dzp solid lipid nanoparticles (SLNs) as well, with the purpose of achieving Dzp prolonged release in suppositories.^9^

Instability is one of the most important challenges with SLNs. In the present study, Dzp SLNs were freeze-dried to increase the stability. Direct freeze-drying of SLNs may cause some problems including aggregation of particles and particle size enlargement. To overcome these restrictions, based on previous experiences, mannitol was used as a cryoprotectant for the lyophilization process.^10,11^

Dzp absorption profile after different administration routes is one of the other fields that has been investigated by many researchers. Suksiriworapong et al^12^ tried to solve limited rectal absorption of Dzp by preparing polymeric micelles drug delivery system. Galletly et al^13^ compared Dzp mixed micelles with Dzp in propylene glycol, and midazolam, they found venous morbidity was 17% for Dzp mixed micelles, 26% for midazolam, and 90% for Dzp in propylene glycol. Dzp mixed micelle formulation was suggested as a preferable alternative to the standard formulations.

There are various studies that have also been focused on designing novel drug delivery systems like Dzp nasal delivery to enhance brain delivery of the drug. A case in point, is a new nasal drug delivery system of Dzp, that has been developed with a natural mucoadhesive agent from fenugreek *(Trigonella foenum-graecum*). This patient-friendly, needle-free dosage form may replace Dzp injections in the future.^14^ Efforts on preparing an oral patch of Dzp, composed of the outer mucoadhesive carbopol 934 region, central drug region, and Tegaderm backing film, have been made as well.^15^

On the basis of the literature reviewed, it was realized that various scientific studies have been conducted to beat the restrictions of Dzp therapy and presumably, SLNs could have the opportunity to be the desirable carrier based on the numerous pros which were discovered by the researchers over the past decades.^16^ The aim of the present research was to improve the delivery of Dzp using nanotechnology. *In vitro* evaluations and *in vivo* distribution of Dzp-SLNs after intraperitoneal (IP) and oral administration (PO) to Wistar rats were investigated as well.

## Materials and Methods

### Materials

Diazepam base (Cambrex, Italy), glycerol monostearate (GMS), stearic acid (SA), and sodium hydroxide (Sigma Aldrich, Germany), cholesterol (CHOL), Tween 80, ethanol, acetone, and mannitol (Merck, Germany).

### Methods

#### UV detection of diazepam

Maximum absorbance wavelength (λ_max_) for Dzp was determined using Shimadzu UV–vis, 2100, Japan, spectrophotometer. In addition, phosphate buffer solution (PBS, pH: 7.4) was used as blank. The calibration curve of Dzp was plotted for concentrations in the range of 2-10 ppm. The plotted calibration curve was used for* in vitro* studies.

#### Preparation of solid lipid nanoparticles

The high shear homogenization technique was selected for SLNs preparation.^17^ Three different types of lipids with the possible capability of forming hydrogen bonds with Dzp for developing a sustained-release drug delivery system were selected as carriers including CHOL, SA and GMS. Among different kinds of surfactants that were reported in publications, Tween 80 was selected as a nonionic surfactant with concentrations of 0.5-1% w/w. Drug concentrations were 0.12 and 0.24% w/w in different preparations. The hot oily phase was prepared by heating the lipids in ethanol and acetone. Following this, the drug was added to the oily phase. The aqueous phase was prepared by adding the surfactant to water. Then the hot oily phase was added to the aqueous phase under homogenization. The homogenization time is a determining factor in the characteristics of particles. Hence, based on the obtained results, 10 minutes homogenization duration was selected as the best condition, after examining 5, 10, and 15 minutes, and the amount of ethanol, acetone, and water were fixed in the volumes of 3, 1 and 25 mL, respectively. The nanoparticles were being formed while the mixture of aqueous and oily phases was cooling down to room temperature under homogenization followed by sonication. At first, nano lipid carriers were developed without drug to justify independent variables. In this step, seven carriers were prepared under reported conditions in the Table 1. In all presented carriers, homogenization and sonication time were fixed at 10 minutes and concentration of Tween 80 was 0.5% w/w. In order to evaluate the effects of increasing surfactant amount, percentage of tween was increased to 1% w/w in (S1-2, S3-2, S4-2).

**Table 1:** Composition of S1-S7 formulations without drug

**Formulation No.**	**Type and amount of lipid(s)** **%w/w**	**Visual stability results, during 2 weeks**
S1	CHOL, % 1	Stable
S2	SA, % 1	Unstable
S3	GMS, % 1	Stable
S4	GMS, % 0.5	Stable
	SA, %0.5	
S5	GMS, %0.5	Stable
	CHOL, %0.5	
S6	CHOL 0.5 + SA 0.5	Stable
S7	GMS 0.33	Unstable
	CHOL 0.33	
SA 0.33

* Optimized formulations.

In the next step of the study, the drug was added to the optimized carriers, and drug-loading efficiency was evaluated.

Table 2, demonstrates the designed and prepared formulations.

**Table 2:** Composition of formulations containing drug

**Formulation No.**	**Type and amount of lipid(s) %w/w**	**Drug amount %w/w**
SLN1	CHOL 1	0.12
SLN2	CHOL 1.4	0.12
SLN3	CHOL 1.4	0.24
SLN4	GMS 0.7+SA 0.7	0.12
SLN5	GMS 1.4	0.12

In all Dzp-SLNs which were mentioned in Table 2 concentration of surfactant was 1% w/w. Moreover, for homogenization and sonication, 10 minutes was applied. Prepared samples were evaluated by measuring particle size, zeta potential, and polydispersity index (PDI) as well as drug-loading efficiency.

#### Freeze-drying

Freeze-drying of selected SLNs was done to increase the stability of particles. Mannitol was used as cryoprotectant of the lyophilization process to prevent the probable aggregation of particles during freeze-drying.

#### Particle size determination

Nanoparticles were evaluated before and after freeze-drying, with respect to their size, PDI, and zeta potential using dynamic light scattering (DLS) instrument and Zetasizer ZS (Malvern Co., UK).

#### Drug-loading efficiency

Drug-loading efficiency (%LE) was determined by the reverse method. For this purpose, the prepared formulations were centrifuged at 20000 rpm for 45 minutes at -4°C. Nanoparticles precipitated and free drug remained in the supernatant. This supernatant was evaluated and the amount of the free drug was detected by UV detector. In the end, LE% was calculated using the following equation.

$$LE(\%)=\left(\frac{W_{Initial\;drug}-W_{Free\;drug}}{W_{Initial\;drug}}\right)\times 100$$

^18^

#### Drug release study

Release study was performed applying dialysis sack method using Spectra/Por^®^ dialysis membrane which had 12 000– 14 000 Mwt cutoff. In the beginning, 5 mL of the prepared formulation was placed in a dialysis membrane and immersed in 400 mL of PBS (pH 7.4). Then 4 mL of samples around the dialysis sack were withdrawn in the desired time intervals and 4 mL fresh PBS was added to the release vessel. Drug concentration was measured and analyzed by the UV detector for each sample. Release study was carried out before and after freeze-drying to ensure that lyophilization did not cause a significant burst effect. In order to compare the release profile of the free drug and the loaded drug, the free Dzp release test was executed as well.^19-21^

#### Morphology studies

Morphology of the nanoparticles was characterized by scanning electron microscopy (SEM) using Philips XL30, Almelo, Netherland’s instrument. The atomic force microscopy (AFM) photographs were taken after freeze-drying, using NT-MDT Spectrum Instrument.

#### Differential scanning calorimetry (DSC) studies

The thermotropic properties and possibility of forming hydrogen bonds between Dzp and lipids, were evaluated by differential scanning calorimeter, (METTLER TOLEDO, USA). Samples of about 5 mg were sealed in 50 μL aluminum pans at a heating rate of 10°C/min throughout the analysis. Empty aluminum pans were used as references and the whole thermal behaviors were studied under a nitrogen purge.^9^

### In vivo studies

Adult male Wistar rats weighing 150-180 (g) were obtained from the animal house of Experimental Medicine Research Center of Tehran University of Medical Sciences (TUMS). The animals were allowed to feed with a standard pellet diet and water ad libitum at 20-25°C under a 12-hour light/dark cycle. Food was withdrawn one day before the experiment, but water continued to be provided. All animal handling and experiment protocols complied with the guidelines of the Laboratory Animal Centre of the University of Tehran.

Ninety-six rats were divided into 4 groups of twenty-four. Group A received Dzp (31 µg PO), group B received 2.015 mg Dzp lyophilized SLNs containing 31 µg Dzp by oral route, group C was given 31 µg Dzp by intraperitoneal injection (IP) and finally, group D received 2.015 mg of Dzp SLNs IP. At the next stage, rats were anesthetized by a cocktail containing a mixture (1:1 v/v; 1 ml/kg body weight) of xylazine 2% (10 mg/kg) and ketamine 10% (50 mg/kg) intraperitoneal (IP), and blood samples were collected from the animal hearts 8, 12 and 24 hours after the treatment protocol. All gathered blood samples were analyzed using High-performance liquid chromatography (HPLC). For each time point, eight animals were studied.^22-26^

## Results and Discussion

### UV detection of Dzp

The λ_max_ of Dzp was determined at 231 nm, and this wavelength was selected for Dzp detection in the *in vitro* studies.

### Calibration curve

Dzp calibration curve in PBS was plotted for the concentrations between 1-10 ppm. For this concentration range, R^2^ was equal to 0.9998.

### Particle size studies

The particle size for ten designed formulations is reported in Table 3. They were in the range of 147-483 nm. S2 and S7 formulations were unstable after 2 weeks and phase separation was observed, therefore, the study was conducted with other formulations. The effect of increasing the Tween 80 percentage on the particle size of the formulations was evaluated on S1, 3, and 4 and they named S1-2, S3-2, and S4-2. In these formulations, percentage of Tween 80 was increased to 1%.

**Table 3:** Particle size results for the SLNs before drug loading

**Formulation No.**	**Particle size nm**
S1	223
S2	162
S3	221
S4	167
S5	376
S6	483
S7	208
S1-2	147
S3-2	211
S4-2	223

### Zeta potential and PDI evaluation of S1, S3, and S4

The zeta potential and PDI evaluations of S1-2, S3-2, and S4-2 were done using the Malvern system. The results were reported -18.1 mv, -13.9 mv, and -14.6 mv for S1-2, S3-2, and S4-2 respectively. Previously, researchers discovers that GMS is a fatty acid ester that may be expected to impart a negative surface charge on the lipid particles.^27^ Subsequently, the PDI of the mentioned SLNs were determined 0.239 for S1-2, 0.239 for S3-2, and 0.508 for S4-2. Particle size, zeta potential, and PDI investigations were repeated for the drug-loaded formulations and results are reported in Table 4.

**Table 4:** Results of the prepared formulations with drug

**Formulation No.**	**Type and amount of lipid** **(%w/w)**	**Type and amount of surfactant** **(%w/w)**	**Drug amount** **(%w/w)**	**Homogenization** **(min)**	**Sonication** **(min)**	**Particle size** **(nm)**	**%Drug loading efficiency**
SLN1	CHOL 1	Tween 80 1	0.12	10	10	267	19.82
SLN2	CHOL 1.4	Tween 80 1	0.12	10	10	195	25.08
SLN3	CHOL 1.4	Tween 80 1	0.24	10	10	135	23.9
SLN4	GMS 0.7 + SA 0.7	Tween 80 1	0.12	10	10	605	56.13
SLN5	GMS 1.4	Tween 80 1	0.12	10	10	130	75.38

After further studies on formulation No. 5 including changing the amount of drug, it was found that the presented production conditions are good enough and the quality parameters were evaluated for this formulation. After freeze-drying of the formulation No. 5, particle size and zeta potential measurements were assessed again to ensure that significant size enlargement did not happen. The zeta potential changed to -16.8 mv and the size of particles was 205 nm for lyophilized formulation.

### Drug release profile

**Figure 1:**
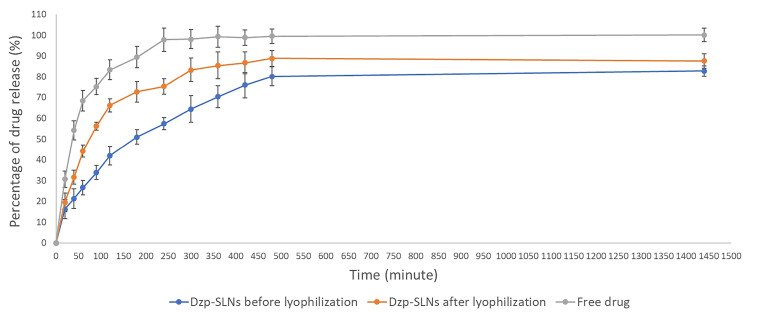
Drug release profiles of free drug, DZP SLNs before and after freeze drying.

The release profiles of the loaded drug in SLNs before and after lyophilization in comparison with the free drug are presented in Figure 1. The free drug rapidly passes through the membrane. After 80 minutes, more than 75% of the dissolved drug was found in receptor phase. Dzp SLNs showed a sustained-drug-release profile. More than 70% of the loaded drug was found after 350, and 450 minutes for SLNs before and after lyophilization, respectively. Total percentage of drug release for lyophilized SLNs was more than initially prepared SLNs.

Although mannitol was used to minimize the stress of lyophilization process on the SLNs, freeze-drying in vacuum stage, caused stress to the particles which might result in porosities on SLNs. Therefore, the differences between lyophilized and non-lyophilized SLNs, are reasonable.

### Morphology studies

**Figure 2:**
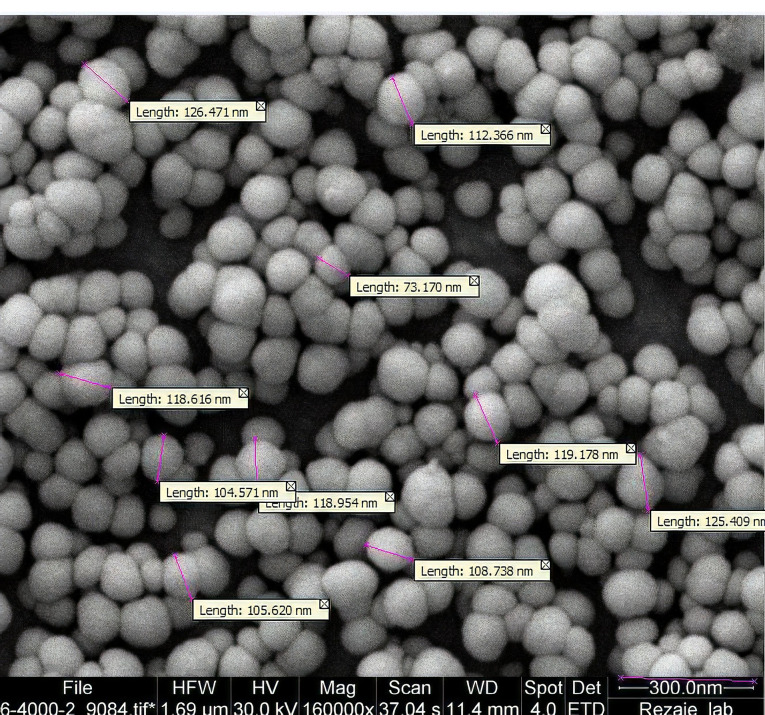
SEM picture of lyophilized nanoparticles.

SEM pictures confirmed the size of particles that had been detected by DLS. Figure 2 shows the SEM photograph of lyophilized Dzp SLNs. Based on the photo all particles are spherical in shape.

**Figure 3:**
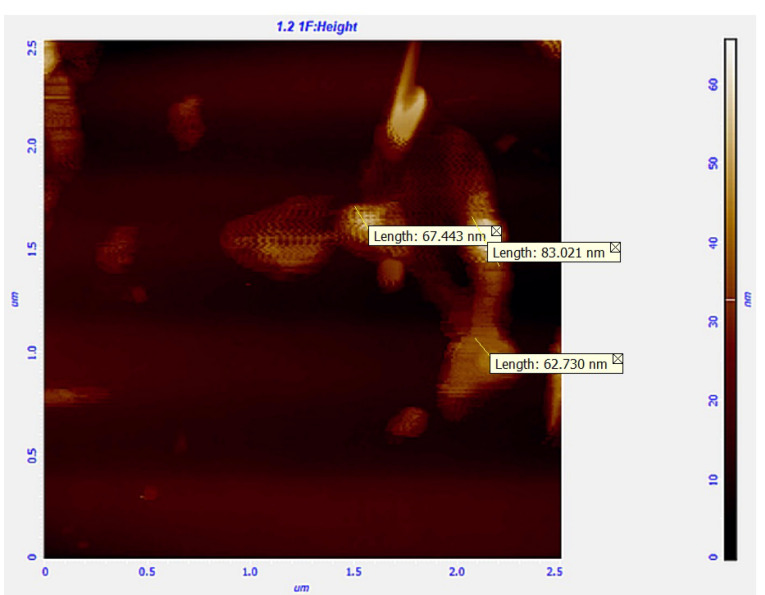
AFM picture of lyophilized nanoparticles.

AFM technique was used to evaluate the shape of particles, and surface characteristics as well as size. Figure 3 indicates the AFM photo, in which the spherical shape of the particles is demonstrated.

### DSC studies

**Figure 4:**
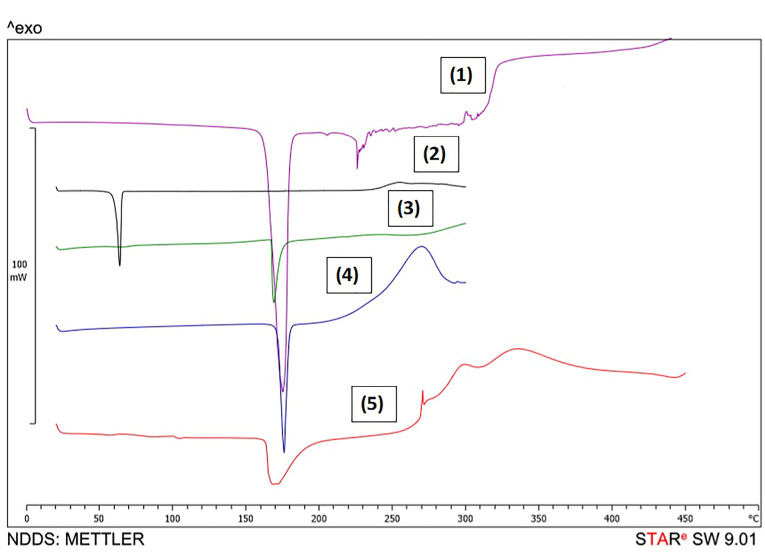
DSC thermograms in which (1) is mannitol, (2) is GMS, (3) is physical mixture of materials, (4) is diazepam and (5) is lyophilized SLNs of diazepam.

DSC was carried out to investigate the probability of forming hydrogen bond(s) between GMS and Dzp. The result which is shown in Figure 4, proved that melting points of these compounds changed after SLN formation. Based on these outcomes, we hypothesized the formation of hydrogen bonding which can cause sustained-drug-release profile. Previous studies confirmed the probability of hydrogen bond forming by the same lipid carriers for some other Active Pharmaceutical Ingredients (APIs) including curcumin as well as amikacin and ampicillin.^28,29^

### In vivo studies

**Figure 5:**
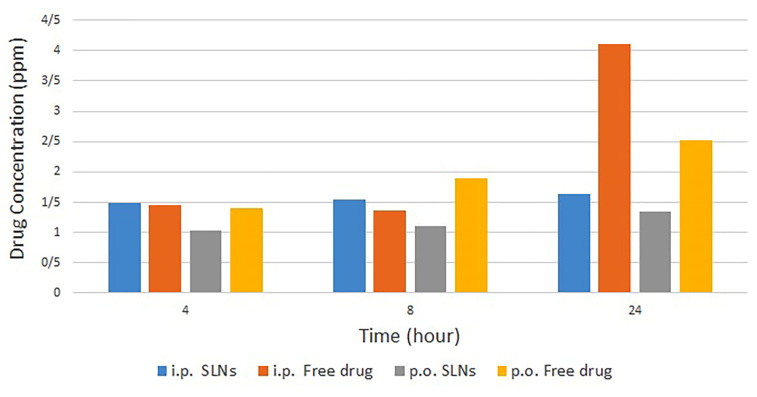
The blood drug concentration profile comparison between four study groups.

According to the findings which were obtained from HPLC and presented in Figure 5, it seems that for the free drug, the blood concentrations were higher in each sampling time in comparison with SLNs. For samples taken at 4 and 8 hours after administration, the achieved data can be the result of sustained-drug-release profile. However, for 24 hours samples in which more than 85% of the drug was released, the lower blood concentrations for SLN samples, illustrate that the drug may be distributed in other tissues of the body and probably in the brain. Clearly, more studies need to be carried out to ensure drug distribution profile. As a recommendation, radioisotope linking could help to track the drug in the body.

Based on this research, it seems that the application of SLNs could help us to inhibit the excretion of Dzp from the brain and increase drug concentration in the site of action. The drug concentration profile in blood after oral administration demonstrated more significant differences by changing free drug to SLNs. In IP administration, the most significant difference refers to 24 hours samples.

## Conclusion

As reported by research findings, it can be concluded that loading Dzp into SLNs could be an appropriate approach for Dzp delivery with fewer side effects due to increased administration intervals in the future. Moreover, it appears that the prepared SLNs, could be suitable semi-finished products, for using in the different dosage forms and subsequently, diverse routes of administration such as intravenous, intranasal, and oral.

## Ethical Issues

The protocol of this experimental study was approved by the Ethical Committee of Tehran University of Medical Sciences (IR.NIMAD.REC.1397.328) and performed in accordance with the ethical guidelines of animal studies, and the International Guiding Principles for Biomedical Research Involving Animals (1985).

## Conflict of Interest

Authors declare no conflict of interest.

## Acknowledgments

Research reported in this publication was supported by Elite Researcher Grant Committee under award number [977099] from the National Institutes for Medical Research Development (NIMAD), Tehran, Iran.

Authors would like to give thanks to Dr. Sanaz Ghaffari and Dr. Taraneh Gazori for the English polishing of the manuscript.
